# Overexpression of the Oncogenic Variant (KLF6-SV1) in Young NPC Patients and Correlation with Lack of E-Cadherin

**DOI:** 10.1155/2018/9654067

**Published:** 2018-04-19

**Authors:** Saoussen Debouki-Joudi, Sonia Mhirsi, Nehla Mokni-Baizig, Nihel Ammous-Boukhris, Hayet Mhamdi, Said Gritli, Raja Mokdad Gargouri, Mohamed Nejib Marzouki

**Affiliations:** ^1^Laboratoire d'Ingénierie des Protéines et des Molécules Bioactives, Institut National des Sciences Appliquées et de Technologie (INSAT), Tunis, Tunisia; ^2^Institut de Carcinologie Salah Azaiz, Tunis, Tunisia; ^3^Laboratoire de Biotechnologie Moléculaire des Eucaryotes, Centre de Biotechnologie de Sfax, Université de Sfax, Sfax, Tunisia

## Abstract

**Purpose:**

The transcription factor Krüppel-like factor 6 (KLF6) regulates various cellular functions, such as metabolism, cell proliferation, and differentiation. KLF6 plays a key role in the development and progression of multiple human cancers.

**Methods:**

Fifty primary biopsies and 10 normal nasopharyngeal mucosae were used to analyze by RT-QPCR the expression and the copy number of wtKLF6 and the spliced variants (KLF6-SV1, KLF6-SV2, and KLF6-SV3) in Tunisian patients with nasopharyngeal carcinoma. The expression analysis of E-cadherin and cyclin D1 was conducted by RT-QPCR and Western blot, respectively.

**Results:**

The wtKLF6 was significantly downexpressed in tumors compared to normal tissues (*p* = 0.0015), whereas KLF6-SV1 and KLF6-SV2 were overexpressed in tumors compared to wtKLF6 and KLF6-SV3 (*p* < 0.0001). Copy number variation was reduced in tumors compared to normal tissues (*p* = 0.0071). Interestingly, KLF6-SV1 is associated with the juvenile form (*p* = 0.0003) which is more aggressive than the adult form of NPC. Furthermore, the oncogenic variant KLF6-SV1 was overexpressed in tumors lacking the expression of E-cadherin (*p* = 0.0022) suggesting its role in metastasis and tumor progression. The wtKLF6 is associated negatively with cyclin D1 in tumor tissues (*p* = 0.048).

**Conclusion:**

The wtKLF6 was downexpressed in contrast with the oncogenic variants. Overexpression of KLF6-SV1 is associated with young patients, and loss of E-cadherin suggests that this variant correlated with the aggressiveness of NPC.

## 1. Introduction

Nasopharyngeal carcinoma (NPC) belongs to head and neck malignant disease with a particular geographical distribution over the world [1]. In Tunisia, NPC represents the most frequent head and neck cancer with a bimodal pattern of age distribution with an annual incidence of about 4 cases per 100,000 persons [1, 2]. There are several clinical and biological characteristics which are specific to the North African patients, especially the two peaks of frequency according to age at diagnosis, the first one around 50 and the second below 30 (20 to 25% of cases). However, in the endemic regions of South-East Asia, there is only one major peak of incidence at about the age of 50 [3–4]. The etiology of the development and progression of NPC is multifactorial such as geographic areas, environmental exposure, diet, Epstein-Barr virus, genetics, and epigenetic factors [5]. Several efforts have been made to identify markers for diagnosis, prognosis, and/or therapy in different types of human cancer including NPC. In this context, we focus on the Krüppel-like factor 6 gene that has been investigated in several cancers but not in NPC except the study of Chen et al., reporting mutation screening of the KLF6 gene in Asian patients [6].

Krüppel-like factor 6, also called KLF6/Zf9/CPBP, is a member of the Krüppel-like family of zinc finger transcription factors. The human KLF6 gene maps on the chromosome 10p15, and the coding sequences are at four exons separated by three introns [7]. Transcription from this gene potentially produces the wild-type and three alternatively spliced isoforms isolated *in vivo*, named KLF6-SV1, KLF6-SV2, and KLF6-SV3 [8]. KLF6 is ubiquitously expressed and involved in cell development, differentiation, proliferation, and apoptosis by interacting with several target genes [9]. In fact, it interacts with DNA through its COOH-terminal domain represented by three zinc fingers, and it was suggested that it could control cell cycle progression through the activation of p21 in a p53-independent manner [10]. KLF6 is largely involved in other functions as both transcriptional activators and repressors such as maintenance of pluripotency, development, growth-related signal transduction, cell proliferation, apoptosis, angiogenesis, skeletal muscle development, and cardiovascular development [8, 9]. In addition, KLF6 can mediate the inhibition activity of cyclin D1/cyclin-dependent kinase leading to growth inhibition [11].

Some studies reported that somatic mutations and LOH occurred frequently in different malignancies such as hepatocellular carcinoma, prostate cancer, glioblastoma, and colorectal cancer [12–14].

Based on the literature data and on the best of our knowledge, there is no previous study investigating the expression of wtKLF6 and its spliced variants at mRNA level as well as the copy number variation in NPC. Thus, the aim of this study is to determine the copy number variation and the expression levels of wtKLF6 as well as of the three spliced variants KLF6-SV1, KLF6-SV2, and KLF6-SV3 in Tunisian patients with NPC. Furthermore, correlation between the expression of wtKLF6 and the spliced variants with their target, namely, the CDH1 encoding E-cadherin and cyclin D1, was assessed.

## 2. Materials and Methods

### 2.1. Patients

Primary NPC biopsies were collected from 50 patients who underwent resection before any treatment at Salah Azaiz Hospital of Tunis (Tunisia). The age of the patients ranged from 14 to 85 years (mean 45.85 years), and the sex ratio was 2 : 1 of men : women. The histological type was determined on tissue sections according to the World Health Organization criteria, and all patients enrolled in this study have tumors of undifferentiated type (UCNT). In addition, 10 histological normal hyperplasia tissues were collected from patients with clinical symptoms indicative of NPC, but nasopharyngeal biopsies did not show tumor cells. These specimens were used as controls. All patients gave informed consent prior to specimen collection according to institutional guidelines. The study was performed in accordance with the ethical standards of the revised Declaration of Helsinki (October 2013).

### 2.2. Extraction of DNA, RNA, and Proteins

Total DNA, RNA, and protein from fresh-frozen tissues were extracted by a TRIzol reagent according to the protocol provided by Invitrogen. The concentration and the purity of the DNA (treated by RNAse from Fermentas) and RNA (treated by DNAse from Invitrogen) were measured by a Nanodrop ND-1000 UV/Vis spectrophotometer (Thermo Fisher Scientific), and DNA and RNA were stored at −20°C and −80°C, respectively, for further use.

### 2.3. cDNA Synthesis and RT-QPCR

cDNA was synthesized from 300 ng of total RNA in a final volume of 20 *μ*L, using 0.25 *μ*g of oligo dT (Invitrogen), 50 nmol of random hexamers (Invitrogen), 10 nmol of each dNTP (Fermentas), 40 U of RNase inhibitor (Invitrogen), 4 *μ*L of 5x RT buffer, and 200 U of MMLV reverse transcriptase (Invitrogen). The reaction mixture was incubated for 10 min at room temperature, then at 37°C for 50 min, and followed by 70°C for 10 min to inactivate the MMLV. cDNA was used as a template for RT-QPCR in 10 *μ*L reaction mixture containing 1 *μ*L of cDNA, 5 *μ*L of 2x EvaGreen MasterMix (Applied Biosystems), and 2 pmol of each primer.

The primer sequences, product sizes, and annealing temperatures are summarized in Table 1. The thermal cycling conditions consist of 10 min at 95°C followed by 42 cycles of 15 s at 95°C and 1 min at 61°C or 60°C. The specificity of the PCR products was confirmed by melting curve analysis from 60 to 95°C with 0.3% of heating rate. Normal nasopharyngeal tissues were used as calibrators to normalize data to calculate the NRQ by the comparative Ct method [15]. All PCR samples were assessed in triplicate, and the mean values were used for analysis. Relative mRNA levels for each sample were quantified using the Cq approach (fluorescence threshold), normalized to GAPDH mRNA as the standard. Expression of GAPDH as an endogenous reference and expression of the target gene in each sample were normalized to the mean of all Cq (Cq Calib) as described previously [16]. The relative gene expression level was calculated as the normalized relative quantity:

NRQ = 2^ΔCq target^/2^ΔCq GAPDH^, where ΔCq_target_ = CqCalib_target_ − Cq_target_ and ΔCq_GAPDH_ = CqCalib_GAPDH_ − Cq2_GAPDH_.

The targets are wtKLF6, KLF6 spliced variants (KLF6-SV1, KLF6-SV2, and KLF6-SV3), and CDH1 encoding the E-cadherin.

### 2.4. KLF6 Copy Number Variation

The reaction was performed in a 10 *μ*L mixture containing 50 ng of genomic DNA, 5 *μ*L of 2x EvaGreen MasterMix (Applied Biosystems), and 2 pmol of each primer (Table 1). The thermal cycling conditions were as follows: 10 min at 95°C, then 40 cycles of 15 s at 95°C and 1 min at 60°C. The specificity of the PCR products was confirmed by melting curve analysis from 60 to 95°C with 0.3% of heating rate. Each case was analyzed in triplicate, and the mean value was calculated. The relative representation of the KLF6 copy number in each tumor with respect to nontumor tissue is given by the formula 2^−∆∆Ct^, as described previously [15].

### 2.5. Western Blot

Total proteins extracted by a TRizol reagent (Invitrogen) were separated on 10% SDS-PAGE and electro-blotted onto the Hybond-P membrane (Amersham). After blocking with 5% nonfat milk and 0.1% Tween 20 in PBS, the membrane was incubated with anti-cyclin D1 (clone SP4, Spring Bioscience; diluted to 1 : 200) and then with the anti-rabbit antibody coupled to peroxidase (diluted to 1 : 2500, Santa Cruz Biotechnology). The blots were visualized using enhanced chemiluminescence ECL+ (Amersham Biosciences) and photographed by the Molecular Imager VersaDoc (Bio-Rad). The intensity of bands was determined by densitometry using the Molecular Imager VersaDoc (Bio-Rad). Samples were considered positive for cyclin D1 if the densitometry value was <1.

### 2.6. Statistical Analysis

Using the GraphPad Prism 5.0 software, statistical analysis was performed and graphs were generated. Data are expressed as the mean ± standard error of the mean (SEM) or as box plots showing the median and interquartile range (box) and the 5–95 percentiles (whiskers). The nonparametric Mann–Whitney *U* test was used for statistical evaluation of the differences between two independent groups. Correlation analyses were performed using Spearman's correlation test. Contingency was assessed using the chi-square test. *p* < 0.05 was considered statistically significant.

## 3. Results

### 3.1. Expression of wtKLF6 and Its Spliced Variants in NPC

**Figure 1:**
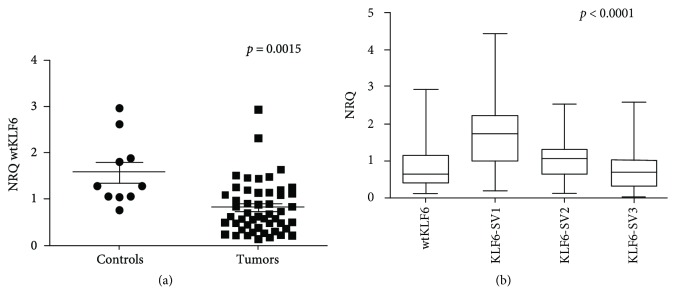
Expression of wtKLF6 in tumors compared to normal tissues. The normalized expression levels of the KLF6 wild-type isoform (a) in tumors versus normal tissues, and (b) the expression levels of the KLF6 wild-type isoform compared to those of the spliced variants KLF6-SV1, KLF6-SV2, and KLF6-SV3.

In this study, 50 UCNT tissues and 10 normal nasopharyngeal mucosae were used to analyze the expression levels of the wtKLF6 and the 3 spliced variants (KLF6-SV1, KLF6-SV2, and KLF6-SV3). We showed that the level of wtKLF6 was significantly lower in tumors than in normal tissues (*p* = 0.0015, Figure 1(a)). The spliced variants KLF6-SV1 and KLF6-SV2 were significantly overexpressed than the wtKLF6 in tumor cases (*p* < 0.0001 and *p* = 0.02215, resp., Figure 1(b)) whereas no difference was seen between wtKLF6 and KLF6-SV3 (*p* = 0.4928).

### 3.2. KLF6 Copy Number Variation and Correlation with Expression Level

**Figure 2:**
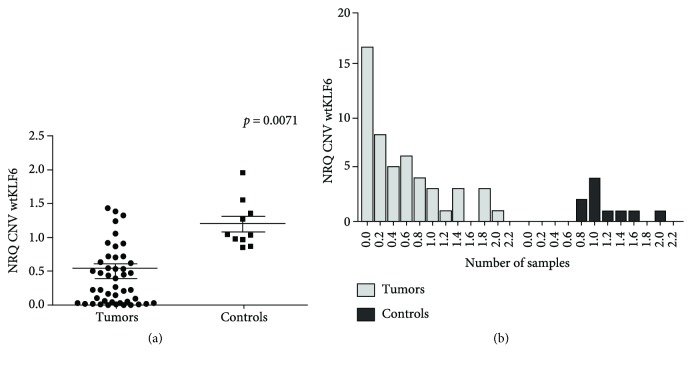
Copy number variation of wtKLF6 in tumors and normal tissues. The normalized copy number variation (CNV) of the KLF6 wild-type isoform in tumors versus normal tissues (a) and the histogram representing the distribution of NRQ values in tumors and controls (b).

Copy number variation (CNV) of the wtKLF6 gene was determined in the 50 tumor samples and 10 normal nasopharyngeal mucosae. We showed that CNV was significantly reduced in tumors compared to normal tissues (*p* = 0.0071, Figure 2(a)). Among the 50 tumor samples, 16 displayed a low NRQ value whereas the NRQ is >2 for only 1 case (Figure 2(b)). Inversely in control samples, the NRQ values vary between 0.8 and 2 (Figure 2(b)).

### 3.3. KLF6-SV1 Is Associated with the Juvenile Form of NPC

**Figure 3:**
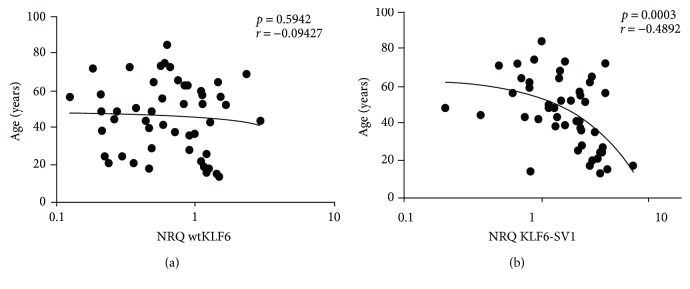
Correlation of wtKLF6 and KLF6-SV1 with age. Association between the expression levels of KLF6-SV1 (a) and wtKLF6 (b) with the age at diagnosis in NPC patients. All correlations were tested using the nonparametric Spearman method.

In our cohort, the mean age is 45.85 and 11 among 50 patients are less than 30 years. Interestingly, expression analysis showed that the oncogenic variant KLF6-SV1 was significantly higher in young patients (under 30 years old) than in adult patients (*p* = 0.0003, Figure 3(b)). No difference in the expression level of the wtKLF6 was observed according to the age of patients (*p* = 0.5942, Figure 3(a)). This finding supports once again that in North African patients, the juvenile form of NPC is characterized by a more aggressive behavior compared to the adult form.

### 3.4. Effect of wtKLF6 and SV1-KLF6 on the Expression of E-Cadherin and Cyclin D1

**Figure 4:**
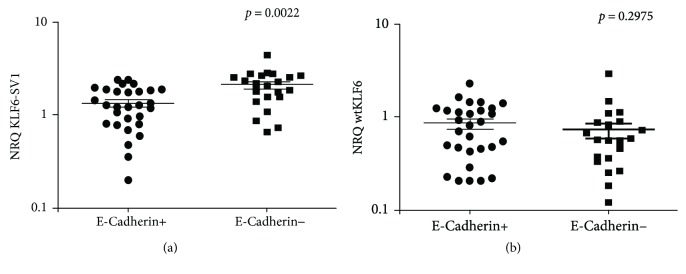
Correlation of wtKLF6 and KLF6-SV1 with the expression of E-cadherin and cyclin D1. Association between the expression levels of E-cadherin with KLF6-SV1 (a) and wtKLF6 (b) in tumor tissues. E-Cadherin+ (NRQ ≥ 0.1) and E-cadherin− (NRQ < 0.1).

**Figure 5:**
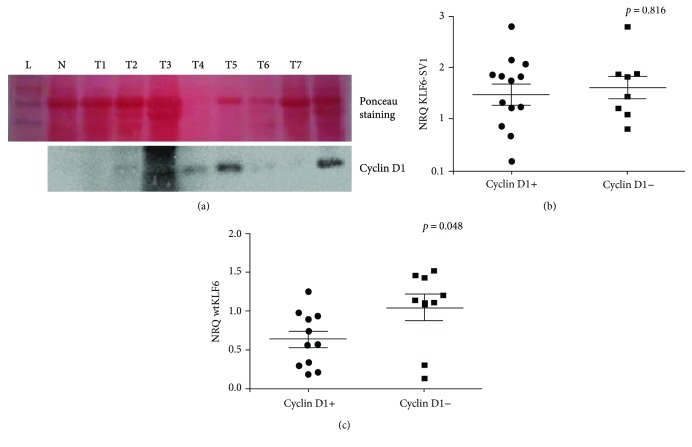
Analysis of cyclin D1 expression and correlation with wtKLF6 and KLF6-SV1. Total proteins extracted from nontumor (N) and tumor samples (T1–T8) were analyzed by Western blot using a monoclonal anti-cyclin D1 antibody followed by HRP-conjugated anti-rabbit IgG, and the signal was revealed by the ECL plus kit (Amersham Biosciences). Ponceau staining was represented in the upper part of (a). Association between the cyclin D1 and the expression levels of KLF6-SV1 (b) and wtKLF6 (c) in tumor tissues. Cyclin D1+ (value densitometry > 1) and cyclin D1− (value densitometry < 1).

In an attempt to validate the effect of KLF6 on its downstream target, we analyzed the expression at mRNA level of the CDH1 encoding E-cadherin. We showed that the expression of E-cadherin was negative in 21 among 50 cases (NRQ values < 0.1) and strongly correlated with high expression of the oncogenic variant KLF6-SV1 (*p* = 0.0022, Figure 4(a)), whereas no association was noted with the wild-type isoform (*p* = 0.2975, Figure 4(b)). This data suggests that KLF6-SV1 leads to a significant decrease in E-cadherin and thus promotes tumor progression and metastasis. Furthermore, we performed Western blot for 20 available samples to investigate the effect of wtKLF6 and the KLF6-SV1 on the expression of cyclin D1. A representative example is illustrated in Figure 5(a). Among 20 samples, 11 were positive for cyclin D1 as the densitometry value was equal to 1. As presented in Figure 5(b), the expression level of the KLF6-SV1 was not correlated with cyclin D1 (*p* = 0.816); however, most of the tumors positive for cyclin D1 displayed low expression of the wtKLF6 (*p* = 0.048, Figure 5(c)) which supports that wtKLF6 negatively regulates cyclin D1.

## 4. Discussion

KLF6 is a Krüppel-like transcription factor ubiquitously expressed and has a key role in human carcinogenesis through regulating several target genes [9, 17]. Indeed, many studies pointed the function of KLF6 as a tumor suppressor because of its ability to reduce cell proliferation through several biochemical mechanisms including regulation of cell cycle, oncogenic products, and apoptosis. Decreased expression of the full length mRNA, mutations, LOH, alternative splicing, and promoter methylation were associated with the development of different human malignancies such as prostate, colorectal, and ovarian cancer and glioblastomas [13, 14, 18, 19]. However, to the best of our knowledge, there is no previous investigation of the KLF6 expression in nasopharyngeal carcinoma except the study of Chen et al. on Chinese patients. Indeed, the authors identify 3 different mutations (Glu75Val, Ser136Arg, and Arg243 Lys) in 3 of 19 NPC tissues [6]. Therefore, in the present study, we investigate the expression levels of the wild-type KLF6 and its oncogenic variants as well as the copy number variation in tumor biopsies and normal nasopharyngeal mucosae of Tunisian patients.

Our results showed that the wild-type isoform of KLF6 (wtKLF6) is significantly downexpressed in tumor tissues compared to normal nasopharyngeal mucosa. It was reported that low expression of the full-length mRNA was detected in glioblastoma tumors which contributes to the activation of the growth of glioma cells [19]. On the other hand, downexpression of KLF6 was also frequently observed in breast and gastric cancers and non-small lung cancer [20–22].

Although KLF6 alternative splicing is present in both normal and cancerous tissues, it was suggested that the ratio between wild-type and alternatively spliced forms have effects on many key processes regulating cancer cell growth and metastasis [23]. In our study, the expression levels of KLF6-SV1 and KLF6-SV2 are significantly higher in tumors than those of wtKLF6; however, no statically difference was observed with KLF6-SV3. Our data is in concordance with that of the previous report showing that alternative KLF6 splicing was a frequent event resulting in the overexpression of KLF6-SV1, KLF6-SV2, and KLF6-SV3 in prostate cancer [24]. In hepatocellular carcinoma and HepG2 cell line, high expression levels of the KLF6-SV2 lead to a significant reduction in cell proliferation associated with apoptosis by activation of p21 (CIP/WAF1) and the proapoptotic Bax gene, mediated by the p53 [25].

Furthermore, Narla et al. demonstrated that high expression levels of the oncogenic variants were associated with advanced tumor stage and grade in prostate cancer [26]. Interestingly, our data showed that KLF6-SV1 correlated significantly with age at diagnosis and that overexpression of KLF6 was more frequently observed in young patients (<30 years) than in adult. This emphasized the aggressiveness of the juvenile form of NPC with frequent distant metastasis and relapse as previously reported [3, 4].

Another part of our study is concerned with the copy number variation of the KLF6 gene in tumor tissues versus normal nasopharyngeal mucosae. We showed that the loss of the KLF6 gene occurred frequently in tumors compared to normal tissues, resulting in low expression level which is in agreement with previous studies [27, 28].

With regard to its role as a transcription factor, KLF6 regulates specific target genes involved in the cell cycle regulation such as p21CIP1/WAF1, cyclin D1, c-Jun, c-Myc, and p53 and in cell invasion such as E-cadherin, MMP-9, and TFPI-2 [8, 9, 29]. Recently, Hsu et al. showed that in oral cancer, ectopic KLF6 expression decreased the migration and invasion of oral cancer cells and suppressed the expression and activities of MMP-9 [30]. Here, we showed that in NPC, lack of E-cadherin expression correlated with high level of the oncogenic variant KLF6-SV1 (*p* < 0.0001). This data suggests that KLF6-SV1 leads to a significant decrease in E-cadherin and thus promotes tumor progression and metastasis. Our data is in line with that of the previous study showing that in breast cancer patients, high level of KLF6-SV1 was associated with increased metastatic potential and poor survival [31]. Furthermore, we found that the expression level of the KLF6-SV1 was not correlated with cyclin D1 (*p* = 0.816), which supports that wtKLF6 negatively regulates cyclin D1 in NPC as previously reported in colon cancer cells [11]. Indeed, Benzeno et al. showed that the levels of cyclin D1 decreased when the level of full-length KLF6 increased, reducing the proliferation and progression of cancer cells.

## 5. Conclusions

In NPC, the wtKLF6 is downexpressed in tumors compared to normal nasopharyngeal mucosae whereas the levels of the spliced variants KLF6-SV1 and KLF6-SV2 were expressed higher than those of the wild-type isoform in tumors. Interestingly, KLF6-SV1 is positively associated with young patients suggesting that this oncogenic variant contributes to the aggressiveness of the juvenile form of NPC. Furthermore, negative association between the expression of E-cadherin and KLF6-SV1 suggests that the oncogenic variant promotes tumor invasion and metastasis in NPC; nevertheless, further studies should be conducted to elucidate the function of KLF6 and its spliced variants in NPC.

## Figures and Tables

**Table 1:** Primer sequences and product sizes of KLF6 (wild type, SV1, SV2, and SV3), CDH1, and GAPDH.

Gene	Primer sequence (5′ to 3′)	Product size (bp)	Annealing T (°C)
wtKLF6 (RT-QPCR)			
Forward	CGGACGCACACAGGAGAAAA	236	61
Reverse	CTCAGCCTGGAAGCCTTTTA		
KLF6-SV1			
Forward	CCTCGCCAGGGAAGGAGAA	237	61
Reverse	CTCAGCCTGGAAGCCTTTTA		
KLF6-SV2			
Forward	TCGGGGAAGCCAGGAGAA	236	61
Reverse	CTCAGCCTGGAAGCCTTTTA		
KLF6-SV3			
Forward	CGGACGCACACAGGTGTT	112	61
Reverse	CTCAGCCTGGAAGCCTTTTA		
KLF6-WT (CNV)			
Forward	CAGTCCTCCAGAGGACACTC	174	60
Reverse	CTCAATTTTCCCGAGCTGACCA		
GAPDH			
Forward	ACCCACTCCTCCACCTTTG	178	60
Reverse	CTCTTGTGCTCTTGCTGGG		
CDH1			
Forward	ACCCACTCCTCCACCTTTG	180	60
Reverse	CTCTTGTGCTCTTGCTGGG		
